# Relationships between the COVID-19 lockdown, socioeconomic factors and acute coronary syndrome hospitalisations in France

**DOI:** 10.1371/journal.pone.0286700

**Published:** 2023-06-07

**Authors:** Rodney Plat, Maria Vasile, François Roubille, Grégoire Mercier

**Affiliations:** 1 Data Science Unit, Montpellier University Hospital, Montpellier, France; 2 Faculty of Medicine, University of Montpellier, Montpellier, France; 3 Cardiology Department, INI-CRT, CHU de Montpellier, PhyMedExp, Université de Montpellier, INSERM, CNRS, Montpellier, France; 4 UMR UA11 IDESP CNRS, University of Montpellier, Montpellier, France; University of Gothenburg: Goteborgs Universitet, SWEDEN

## Abstract

**Introduction:**

Worldwide, the COVID-19 pandemic has been associated with an overall drop in acute coronary syndrome (ACS) hospitalizations. Additionally, there is a well-known association between ACS and socioeconomic status. This study aims to assess the COVID-19 effect on ACS admissions in France during the first national lockdown and investigate the factors associated with its spatial heterogeneity.

**Materials and methods:**

In this retrospective study, we used the French hospital discharge database (PMSI) to estimate ACS admission rates in all public and private hospitals in 2019 and 2020. A negative binomial regression explored the nationwide change in ACS admissions during lockdown compared with 2019. A multivariate analysis explored the factors associated with the ACS admission incidence rate ratio (IRR, 2020 incidence rate/2019 incidence rate) variation at the county level.

**Results:**

We found a significant but geographically heterogeneous nationwide reduction in ACS admissions during lockdown (IRR 0·70 [0·64–0·76]). After adjustment for cumulative COVID-19 admissions and the ageing index, a higher share of people on short-term working arrangements during lockdown at the county level was associated with a lower IRR, while a higher share of individuals with a high school degree and a higher density of acute care beds were associated with a higher ratio.

**Conclusions:**

During the first national lockdown, there was an overall decrease in ACS admissions. Local provision of inpatient care and socioeconomic determinants linked to occupation were independently associated with the variation in hospitalizations.

## Introduction

The current coronavirus 2019 (COVID-19) pandemic poses unprecedented challenges to health care systems. Transitions in the organisation of hospital services and in the use of health care by the population have had a major impact on hospital activities, particularly in the sectors managing noncommunicable diseases [1]. During the first wave of the pandemic, when hospital overload was at its highest, significant drops in the volume of admissions worldwide ranging from 20.2% to 73% were observed for acute cardiovascular diseases [2]. In France, a 30% decrease in the volume of admissions for myocardial infarction (MI) was reported during the first month of the national lockdown, which was implemented from 17 March to 11 May 2020 [3].

However, there are notable territorial disparities in the burden of acute coronary syndromes (ACSs) in France as well as in the impact of the epidemic on the health care system [4,5]. Moreover, the crisis has had a differentiated impact on individuals according to their socioeconomic status, affecting the most disadvantaged individuals more frequently and more severely [6,7]. Additionally, the independent link between social determinants and cardiovascular diseases is well established in developed countries [8–10]. These worrying findings highlight the collateral effects of the pandemic on hospital admissions for ACS and the role that health inequalities may have had. A better understanding of these events is essential to better anticipate them, enabling our health systems to adapt faster to future health crises and ensure the safety of individuals.

### Objectives

We hypothesise that the first lockdown reduced hospital admissions for ACS in France and that this effect was partly dependent on contextual factors related to social inequalities in health. Our objectives were, first, to quantify the trend of admissions in 2019 and 2020 and more specifically during the first national lockdown period in comparison with the same period in 2019 and, second, to investigate the contribution of contextual factors to the variation of these admissions during this period.

## Materials and methods

### Study design and population

In this French retrospective nationwide study, we used data from the French hospital discharge database (PMSI) to estimate weekly rates of admission for ACS. The PMSI is a national database that includes standardised medical information on hospital admissions, such as diagnoses, medical procedures and health care use. We included every admission for adult patients to any public or private hospital for ACS in metropolitan France from 1 January 2019 to 31 December 2020.

### Procedures

**Fig 1:**
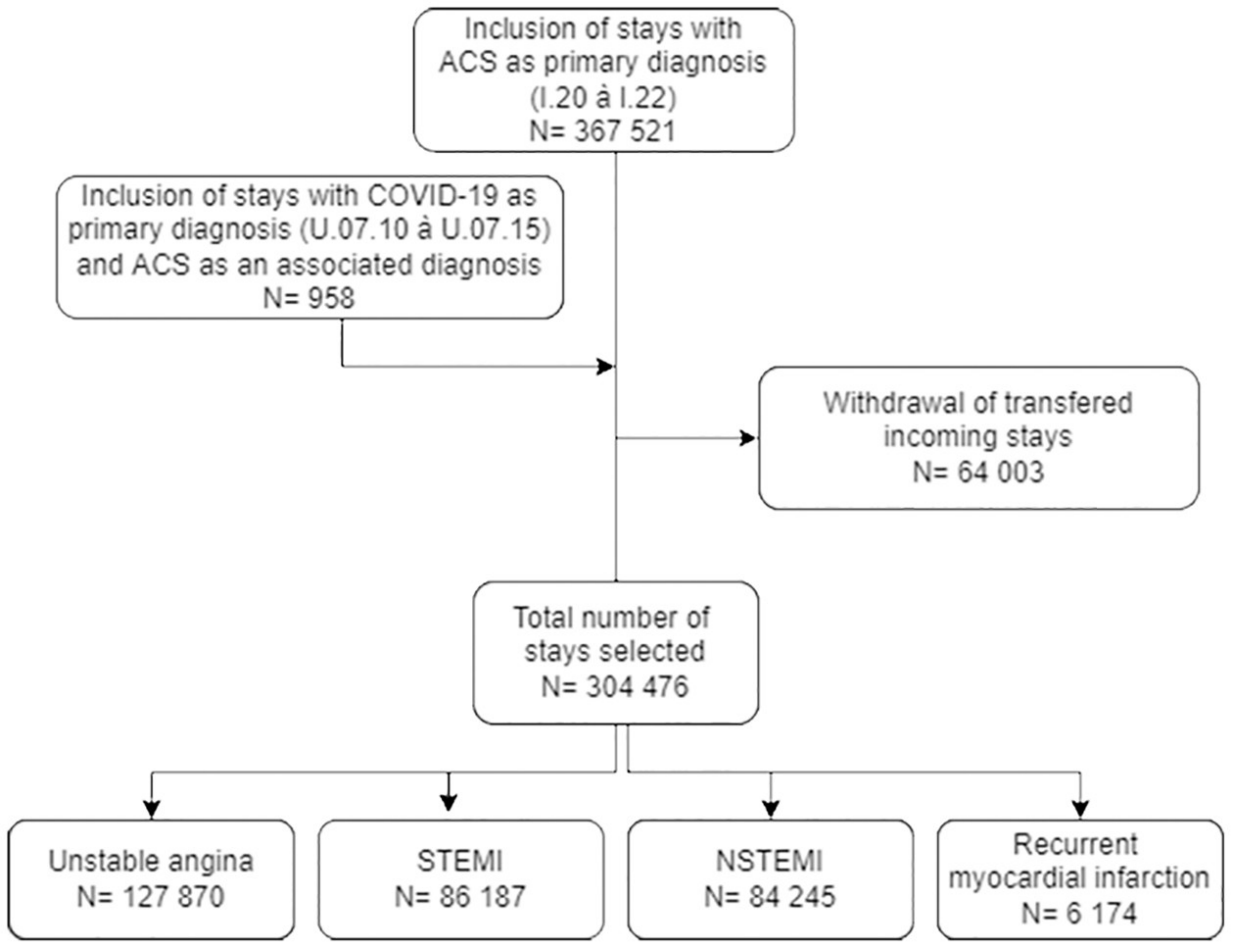
Study flow-chart.

ACS admissions included hospitalisations for unstable angina (ICD-10 code I.20), ST segment elevation myocardial infarction (STEMI, ICD-10 code I.21 except I21.4), non–ST-segment elevation myocardial infarction (NSTEMI, ICD-10 code I.21.4), and recurrent myocardial infarction (ICD-10 code I.22) [11]. To take into account some iterative revisions of the PMSI coding rules published during the crisis and the coding errors that may have resulted, especially in the case of hospitalisation for an ACS associated with a COVID-19 infection, we also included stays with an associated secondary diagnosis code related to COVID-19 (ICD-10 codes U.07.10 to U.07.15) if the ACS ICD code was found at the onset of the hospitalisation. To avoid iatrogenic events and MI occurring during hospitalisation, we excluded patients with a main diagnosis of ACS transferred from another hospital or department (Fig 1).

To investigate the contribution of the factors involved in the variation of admissions during the first lockdown, the multivariate analyses used environmental data aggregated at the county level. The socioeconomic level was measured by the average income, education level, labourers and unemployment rates at the county level provided by the National Institute of Statistics and Economic Studies (INSEE) [12]. Social isolation was measured by the share of single-person households [12]. Socioeconomic determinants also included data on living conditions during the lockdown (decline of financial situation, short time working, teleworking) from the national Epicov study [13].

The supply of inpatient and outpatient care was measured by the density of general practitioners and the density of acute care beds in medicine. Hospital accessibility was also measured by the average travel time between the ZIP code of residence and the ZIP code of the nearest hospital for the patients included in this study, available in the PMSI. To adjust for the local burden of the epidemic, we used the cumulative incidence of admissions for COVID-19 during the first lockdown at the county level. We also included the precrisis global and cardiovascular health condition of populations with the ageing index (i.e., the ratio of the population aged 65 and over to the population under 20), the standardised mortality rate of people aged over 65 years, and the standardised rate of people hospitalised for ACS in 2018 at the county level.

### Statistical analysis

The weekly incidences of admissions for the different types of ACS, as well as for hospital mortality, were estimated at the national level and over the years 2019 and 2020. We conducted a univariate analysis to quantify the first lockdown’s effect on admissions for all types of ACS. Their national cumulative incidence were compared over identical periods, including the first French national lockdown, corresponding to weeks 12 to 19 of 2019 and 2020 (i.e., 18 March to 12 May 2019 and 16 March to 10 May 2020). To address the overdispersion found in the data, a negative binomial regression was used to assess the change in the number of cumulative admissions for the different types of ACS over these two periods (except for recurrent MI, for which there was insufficient data). This regression quantifies the impact of the first confinement on ACS admissions, which is represented by a national cumulative incidence rate ratio (IRR) [3]:

$$\begin{array}{c} National\;cumulative\;incidence\;rate\;ratio\;\left(IRR\right)=\\ \frac{Cumulative\;incidence\;rate\;of\;admissions\;in\;France\;during\;the\;period\;of\;interest,\;in\;2020}{Cumulative\;incidence\;rate\;of\;admissions\;in\;France\;during\;the\;period\;of\;interest,\;in\;2019} \end{array}$$

Using ZIP codes attached to each stay in the PMSI, the IRR for any type of ACS was mapped at the county level, leading to a local cumulative incidence ratio that allowed us to report the impact of lockdown on ACS hospitalisations in each county:

$$\begin{array}{c} Local\;cumulative\;incidence\;rate\;ratio\;\left(IRR\right)i=\\ \frac{Cumulative\;incidence\;rate\;of\;admissions\;in\;country(i)\;during\;the\;period\;of\;interest,\;in\;2020}{Cumulative\;incidence\;rate\;of\;admissions\;in\;country(i)\;during\;the\;period\;of\;interest,\;in\;2019} \end{array}$$

Using linear regression, we conducted univariate and multivariate analyses using counties as statistical units to explore the factors associated with the variation in the local cumulative IRR, which was our dependant variable. Each county-level aggregate environmental data listed in the procedure section was tested univariately with our dependant variable. Multi-collinearity issues were investigated, and each variable associated with the local IRR with a p-value < 0.20 was inserted into the multivariate model. Of the included variables that were no longer significant in the multivariate analysis, the ageing index and the cumulative county incidence of admissions for COVID-19 during lockdown were maintained in the model as they are adjustment variables, reflecting the local burden of COVID-19, as well as the overall and cardiovascular health condition of populations prior to the crisis. Descriptive analyses were performed, reporting continuous data as mean values with standard deviation (SD) and median values with range (quartile 1 and quartile 3).

As the data were spatially distributed, local and global spatial dependence phenomena were screened using Moran’s I and LISA tests. Residual spatial interactions remaining in the linear regression model were searched using Moran’s I test on residuals. Given the persistence of spatial autocorrelation in the errors of the model, a spatial error model (SEM) using queen contiguity was estimated [14]. Its validity was verified by the Hausman test. The analyses were carried out using R studio v4.0.3.

### Ethics statement

This study was approved by the Institutional Review Board of the Montpellier university hospital (IORG number 0009525—I.R.B approval number: 202000600). Since all patient-level data in the PMSI database are anonymized before third parties are given access, informed consent at an individual patient level was not required. The study posed no potential risks to individuals or individual privacy, and was conducted in accordance with relevant international and French regulatory requirements.

## Results

### Admissions and in-hospital mortality rates for ACS in 2019 and 2020

**Fig 2:**
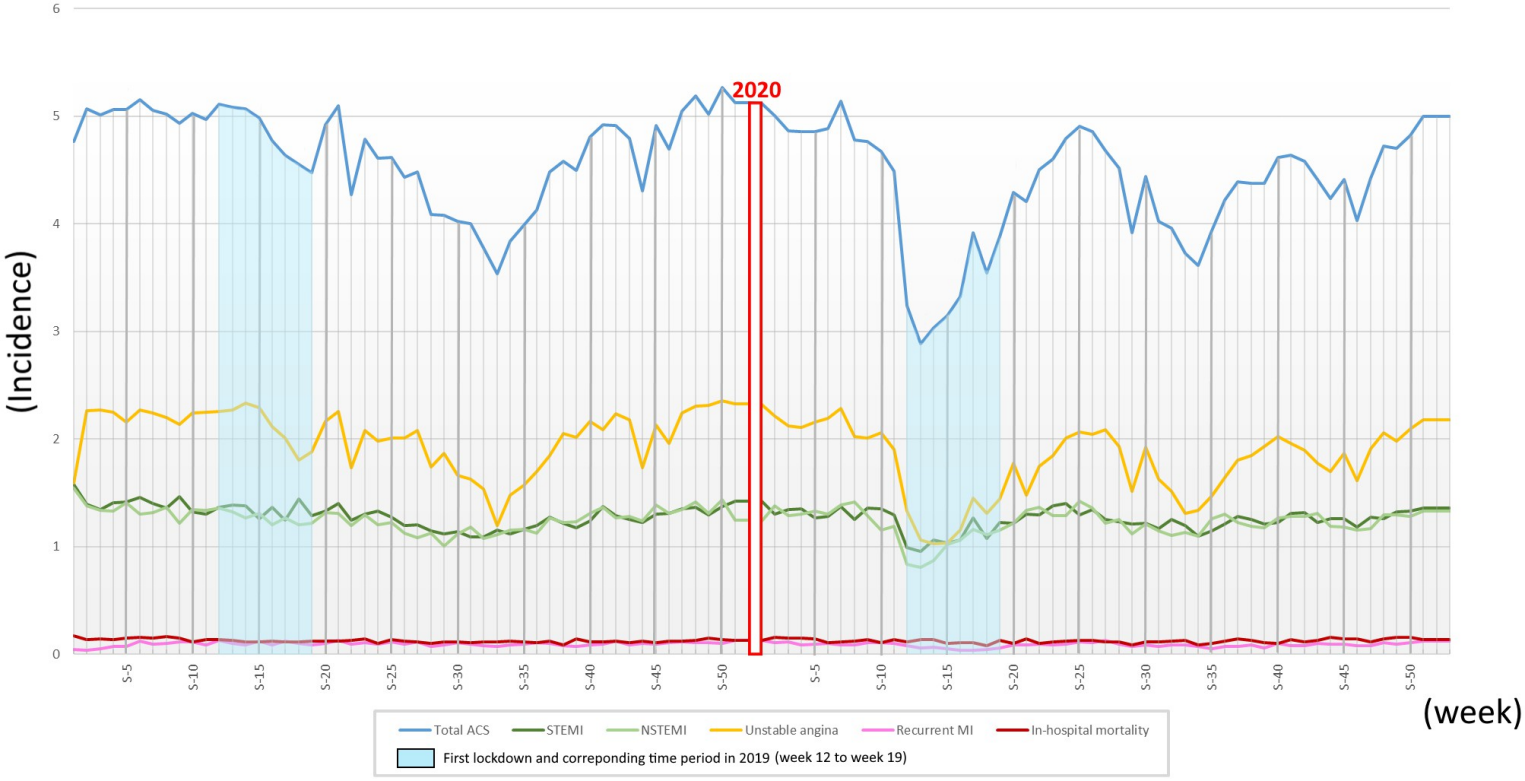
Trends in national weekly incidence of admissions and in-hospital mortality per 100,000 inhabitants.

Fig 2 shows the annual trend of the respective incidences measured. A total of 304,467 stays were included, of which 42,706 occurred during the periods of interest.

During 2019, the average weekly incidence of admissions in France for all types of ACS was 4.68/100,000 inhabitants; 2.03/100,000 inhabitants for unstable angina; 1.30, 1.26 and 0.10/100,000 inhabitants for STEMI, NSTEMI and recurrent MI, respectively; and 0.13/100,000 inhabitants for in-hospital mortality. In 2020, they were equal to 4.34/100,000 inhabitants for all types of ACS; 1.79/100,000 inhabitants for unstable angina; 1.25, 1.22 and 0.09/100,000 inhabitants for STEMI, NSTEMI and recurrent MI, respectively; and 0.12/100,000 inhabitants for in-hospital mortality.

For all types of ACS, weekly admissions peaked in early and late 2019 and 2020, with weekly of approximately 5.0/100,000 inhabitants. During the first lockdown, the incidence ranged from 2.89 to 3.92 hospitalisations/100,000 inhabitants, while the corresponding period in 2019 recorded an incidence ranging from 4.55 to 5.11 hospitalisations/100,000 inhabitants. In the latter part of 2020, the weekly incidence remained, on average, lower than those observed in 2019. These trends are found for all types of ACS, except for recurrent MI, as well as for in-hospital mortality, which appears to be stable overall during 2019 and 2020. The data additionally highlights the presence of a second drop in incidences from weeks 25 to 40 in 2019 and 2020, which could be linked to the well-known summer decrease in incident cases of ACS [15].

### The first national lockdown impact

In France, there was a significant drop in admissions for all types of ACS during the lockdown, accounting for 30% of the volume of hospitalisations recorded over the same time period in 2019 (IRR 0.70 [0.64–0.76], p value < 0.0001). This decline was most noticeable for unstable angina hospitalisations, with a 39% reduction in the number of admissions in 2020 (IRR 0.61 [0.54–0.70], p value < 0.0001). For myocardial infarction, hospitalisations were more modestly affected, with a similar decrease in volumes for NSTEMI (IRR 0.79 [0.71–0.88], p value < 0.0001) and STEMI (IRR 0.80 [0.74–0.86], p value < 0.0001). In-hospital mortality did not vary significantly during the first lockdown (IRR 0.91 [0.79–1.06], p value 0.22).

**Fig 3:**
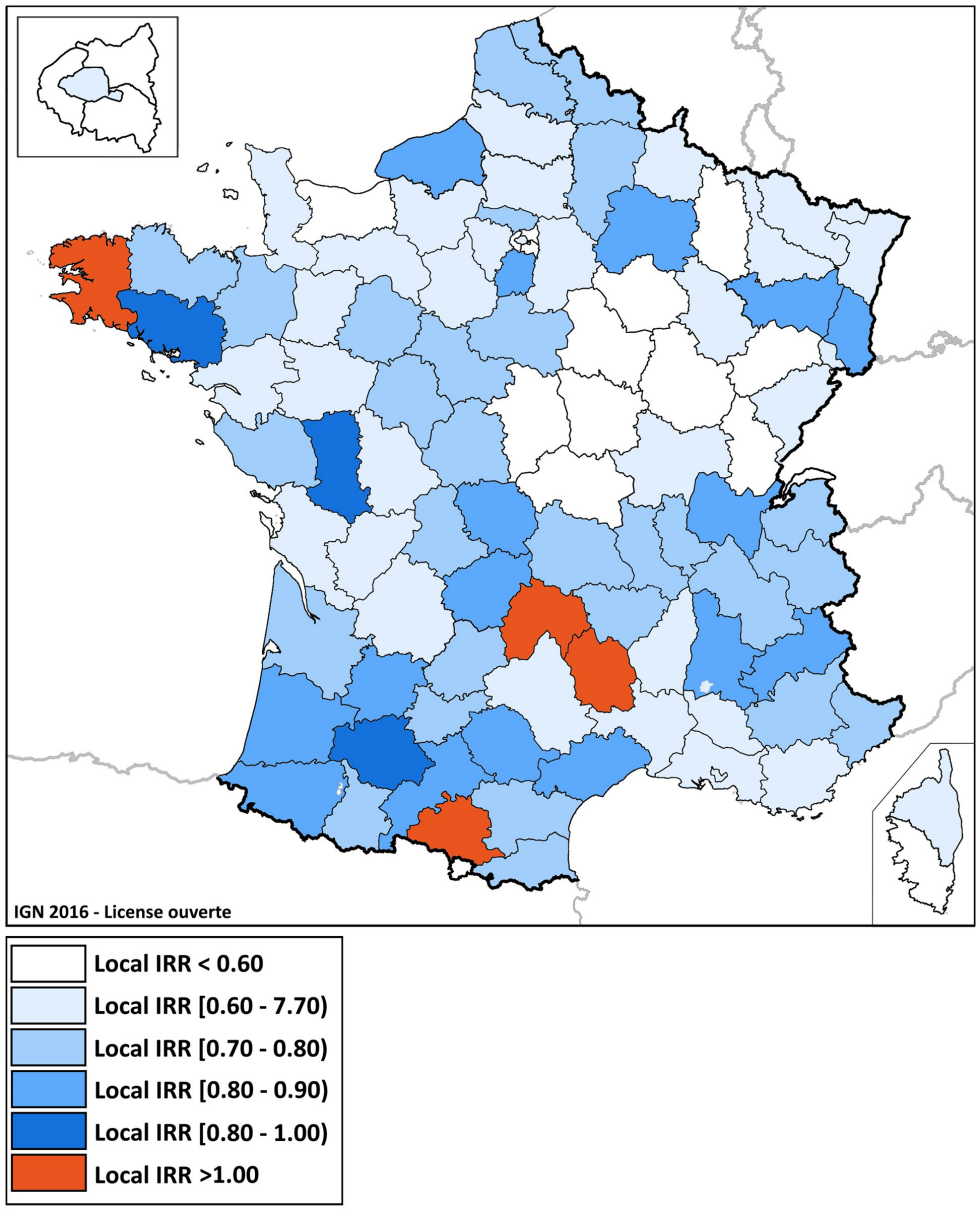
Local IRR of ACS admissions’ territorial variation, from weeks 12 to 19. This figure displays the values of the county cumulative admission Incidence Rate Ratio (Local IRR, which is the ratio of the local cumulative incidence for all types of ACS admissions between weeks 12 and 19 of 2020, to the same cumulative incidence in 2019) for all types of ACS (Background map source: IGN 2016 –Etalab Open license).

Fig 3 shows the county variation of the national admission IRR for all types of ACS, depicting variation in volumes from a decrease of 39% to an increase of 61%. Despite a national trend of a 30% drop in the total number of admissions, four counties recorded a higher number of admissions for ACS in 2020. In contrast, fourteen departments were the most severely affected by the crisis, with relative decreases exceeding 40% over the course of the lockdown.

**Fig 4:**
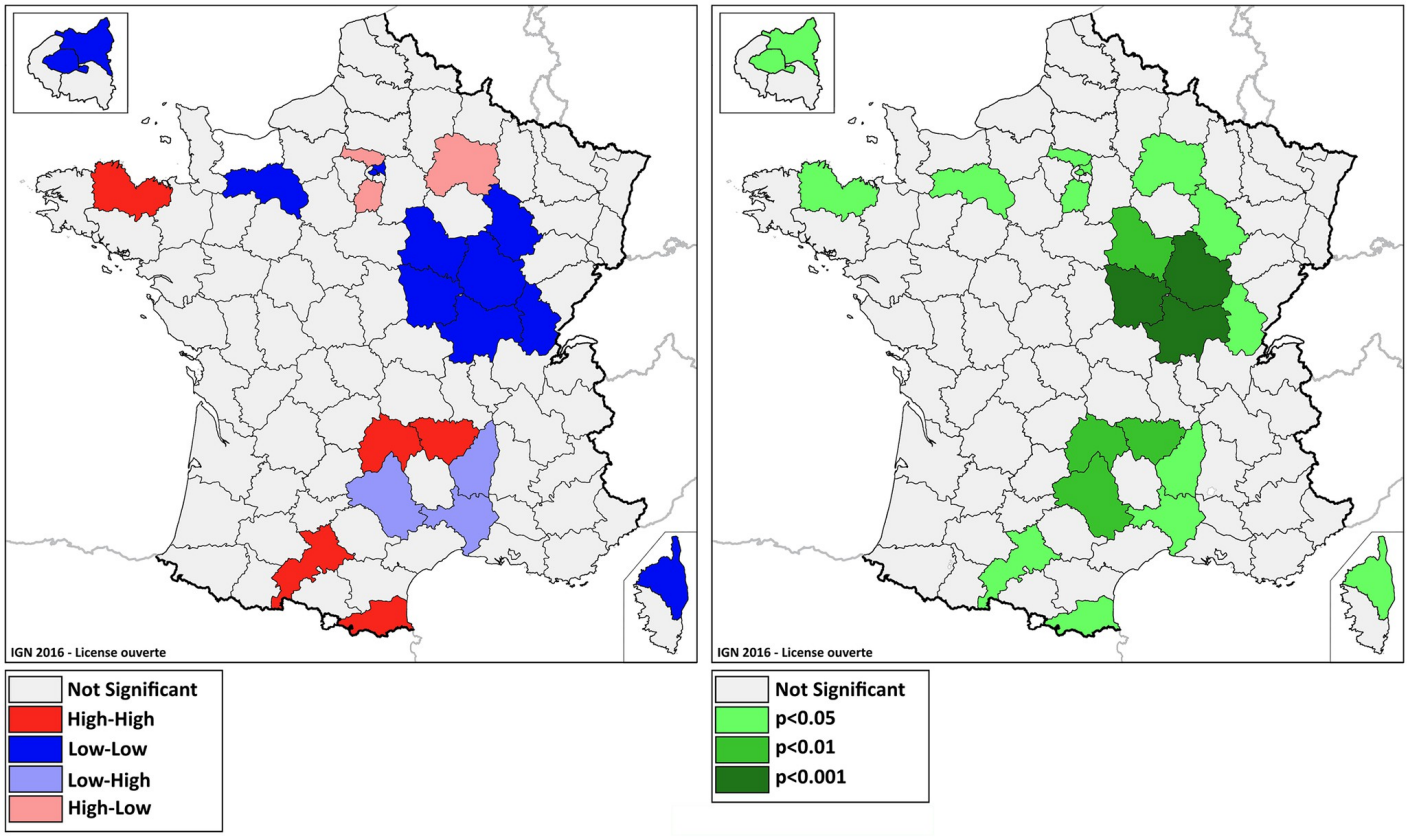
Local spatial autocorrelation clusters (LISA). This figure highlights the countries for which LISA local spatial autocorrelation tests revealed statistically significant clusters within the local IRRs. Significant LISA clusters are combinations of similar or dissimilar values more marked than what might have been observed based on random spatial distribution. These groupings can match up with four types of spatial groupings (high-high, low-low, high-low or low-low). The left panel displays the type of the LISA test clusters. The right panel shows the significance (p-value) of the LISA test clusters. (Background map source: IGN 2016 –Etalab Open license).

Moran’s I test revealed a positive spatial autocorrelation of county IRRs (Moran index 0.24, p value < 0.001). In addition, the local spatial autocorrelation tests LISA found statistically significant clusters of greater decrease or increase in hospital admissions for ACS in 2020. Fig 4 shows their distribution.

### Multivariate analysis

Table 1 presents the mean value and distribution by county of the variables used in this study. Each variable univariably associated with the variation of the IRR with a p value < 0.20 was inserted in the multivariate model. No interactions for the inserted variables were found. The assumptions of the linear model were respected, but a spatial interaction phenomenon was detected in the residuals of the linear model (Moran’s test on the residuals, p value 0.001). The use of an SEM was considered valid (Hausman test p value 0.93) [14].

**Table 1:** Measures of position and dispersion in collected variables.

	Variables	Mean	St. Dev. ^a^	Median	[Q1–Q3]
**Y**	County cumulative incidence rate ratio ^b^	0.72	0.16	0.71	[0.62–0.80]
**Socio-economic data**	Median annual available income per consumption unit, in euros	21395	1672.5	21010	[20415–21908]
	Share of workers in the total workforce, in %	22.2	4.3	22.3	[19.5–25.3]
	Share of individuals whose highest degree is a high school diploma, in the population aged 15 and over not attending school, in %	17.2	1.3	17.1	[16.3–17.9]
	Average annual unemployment rate, %	8.1	1.6	7.9	[7.0–8.9]
	Share of households occupied by a single person, in %	36.2	3.2	36.6	[34.3–37.9]
	Share of people reporting a worsening of their financial situation during lockdown, in %	28.7	3.0	28.7	[26.6–30.5]
	Share of people who have been entirely on part time working arrangement during lockdown, in %	15.2	2.6	14.8	[13.3–17.1]
	Share of people declaring to have exclusively teleworked during lockdown, in %	21.8	9.8	18.8	[15.5–24.3]
**Health care provision**	Density of general practitioners, per 100,000 inhabitants	149.7	27.7	149.5	[132.8–166.0]
	Density of acute care medical beds, per 100,000 inhabitants	229.0	48.8	231.0	[191.8–254.8]
	Average time from place of residence to nearest hospital for included patients, in minutes	20.2	8.8	20.0	[14.2–24.7]
**COVID**	Cumulative county incidence of hospital admissions for COVID-19 during lockdown, per 100,000 inhabitants	130.6	105.0	92.1	[56.9–183.7]
**Overall health**	Standardised mortality rate of over-aged 65s, per 100,000 inhabitants	3725	292.0	3710	[3540–3925]
	Standardised rate of people treated for ACS in 2018, per 100,000 population	157.2	24.8	160.0	[140–170]
	Ageing index	95.4	25.0	92.4	[77.3–114.1]

Table 1 displays the measures of position and dispersion by county of the collected variables

^a^ St. dev: Standard deviation.

^b^ The county cumulative Incidence Rate Ratio is the ratio of the local cumulative incidence for all types of ACS admissions between weeks 12 and 19 of 2020, to the same cumulative incidence in 2019. This variable is the outcome of our model.

Tables 2 and 3 presents the results of the univariate and multivariate analyses. At the county level, the regression models indicate an association between, on the one hand, the rate of individuals whose highest degree is a high school diploma (p 0.003) and the density of acute care beds in medicine (p 0.013) and, on the other hand, the positive variation in the IRR of admissions for ACS. Thus, for each 1% increase in the high school graduate rate, the IRR increased by 4.1%, while the provision of one additional acute care bed per 100,000 inhabitants was associated with a 0.07% ratio increase. Conversely, we found an association between, on the one hand, the rate of individuals who were on short time working during the crisis and, on the other hand, the negative variation in the IRR of hospitalisations for ACS (p 0.008). As a result, for each 1% increase in the short-term working individual rate, the IRR decreased by 1.5%. These results are adjusted by the cumulative incidences of hospital admissions for COVID-19 and the ageing index.

**Table 2:** Results of univariate analysis.

Variables *p-value < 0*.20 * / p-value < 0.05 ** / p-value < 0.01 ***		Univariate regression	
		Coefficient ^a^	St. Dev ^b^
**Socio-economic data**	Median annual available income per consumption unit, in euros	-9.757e-06	9.619e-06
	Share of workers in the total workforce, in %	-0.003461	0.003752
	Share of individuals whose highest degree is a high school diploma, in the population aged 15 and over not attending school, in %	0.03390 ***	0.01179
	Average annual unemployment rate, in %	-0.012066	0.009872
	Share of households occupied by a single person, in %	0.004940	0.005085
	Share of people reporting a worsening of their financial situation during lockdown, in %	-0.010379 **	0.005191
	Share of people who have been entirely on short time working arrangement during lockdown, in %	-0.015447 **	0.006063
	Share of people declaring to have exclusively teleworked during lockdown, in %	-0.001885	0.001630
**Health care provision**	Density of general practitioners, per 100,000 inhabitants	0.0008759	0.000577
	Density of acute care medical beds per 100,000 inhabitants	0.0001655 *	0.000331
	Average time from place of residence to nearest hospital for included patients, in minutes	0.0005683	0.001849
**COVID**	Cumulative county incidence of hospital admissions for COVID-19 during lockdown, per 100,000 inhabitants	-0.0004149 ***	0.000148
**Overall health**	Standardised mortality rate of over-aged 65s, per 100,000 inhabitants	4.232e-05	5.523e-05
	Standardised rate of people treated for ACS in 2018, per 100,000 population	0.0007425	0.000647
	Ageing index	0.0010448 *	0.000638

Table 2 displays the results of the univariate regression between the variables and our outcome, which is the county cumulative Incidence Rate Ratio. Asterisks indicate the level of significance of each link between a variable and the outcome. Each variable univariably associated with the local IRR with a p-value < 0.20 was inserted in the multivariate model.

^a^ Coefficient: Beta regression coefficient. This coefficient corresponds to the degree of in the outcome for every 1-unit of change in the predictor variable being tested. For example, for each change of one unit in a predictor variable significantly associated with the outcome, the IRR will increase or decrease, depending on whether the beta coefficient is positive or negative, respectively. For each change of one unit in a predictor variable significantly associated with the outcome, the ratio will decrease or increase by 1% for each 0.01 point of the beta coefficient.

^b^ St. Dev: Standard deviation.

**Table 3:** Results of multivariate analyses.

**Variables** *p-value < 0*.20 * / p-value < 0.05 ** / p-value < 0.01 ***		**Linear regression (LR)**		**SEM regression (SEM R)**	
		**Coefficient** ^a^	**St.Dev** ^b^	**Coefficient** ^a^	**St.Dev** ^b^
**Socio-economic data**	Median annual available income per consumption unit, in euros				
	Share of workers in the total workforce, in %				
	Share of individuals whose highest degree is a high school diploma, in the population aged 15 and over not attending school, in %	0.0410551 (p 0.002)***	0.013174	0.04091033 (p 0.003)***	0.013937
	Average annual unemployment rate, in %				
	Share of households occupied by a single person, in %				
	Share of people reporting a worsening of their financial situation during lockdown, in %				
	Share of people who have been entirely on short time working arrangement during lockdown, in %	-0.0172224 (p 0.007)***	0.006244	-0.0149843 (p 0.008)***	0.005657
	Share of people declaring to have exclusively teleworked during lockdown, in %				
**Health care provision**	Density of general practitioners, per 100,000 inhabitants				
	Density of acute care medical beds per 100,000 inhabitants	0.0007204 (p 0.041)**	0.000347	0.00078213 (p 0.013)**	0.000317
	Average time from place of residence to nearest hospital for included patients, in minutes				
**COVID**	Cumulative county incidence of hospital admissions for COVID-19 during lockdown, per 100,000 inhabitants	-0.0002955 (p 0.093)*	0.000174	-0.0003159 (p0.091)*	0.000187
**Overall health**	Standardised mortality rate of over-aged 65s, per 100,000 inhabitants				
	Standardised rate of people treated for ACS in 2018, per 100,000 population				
	Ageing index	-0.0009059 (p 0.228)	0.000746	-0.0008510 p(0.251)	0.000742
**Multivariate Analysis parameters**		**Linear regression (LR)**		**SEM regression (SEM R)**	
		**Coefficient** ^a^	**St.Dev** ^b^	**Coefficient** ^a^	**St.Dev** ^b^
	Intercept *(Alpha coefficient of the regression)*	0.2395625	0.251482	0.19318832	0.26730
	Λ *(Errors’ spatial auto correlation coefficient)*			0.329 (p 0.017)**	0.12685
	AIC *(Akaike information criterion)*	-93.179		-96.917	
	R^2^ (Linear *regression*) / pseudoR^2^ (Spatial Error Model regression)	16.9%		27.7%	

Table 3 displays the results of the multivariate regression between the variables and our outcome, which is the county cumulative Incidence Rate Ratio. The shaded variables were not retained in the final model. Asterisks indicate the level of significance of each link between a variable and the outcome. Each variable univariably associated with the local IRR with a p value < 0.20 was inserted in the multivariate model. Of the variables included, the share of people reporting a worsening of their financial situation during lockdown was no longer significant in the multivariate analysis, and was removed from the model. This was also the case for ageing index and the cumulative county incidence of hospital admissions for COVID-19 during lockdown, which were nonetheless maintained in the model as they are adjustment variables.

^a^ Coefficient: Beta regression coefficient. This coefficient corresponds to the degree of in the outcome for every 1-unit of change in the predictor variable being tested. For example, for each change of one unit in a predictor variable significantly associated with the outcome, the IRR will increase or decrease, depending on whether the beta coefficient is positive or negative, respectively. For each change of one unit in a predictor variable significantly associated with the outcome, the ratio will decrease or increase by 1% for each 0.01 point of the beta coefficient.

^b^ St. Dev: Standard deviation.

## Discussion

### Admission trends in 2019–2020 and the impact of the lockdown

During the first lockdown, our study highlights a substantial drop for all types of ACS admissions in France. This decrease was heterogeneous over the French metropolitan territory and seemed to intensify following a line connecting southwestern France to northeastern France. It was also more pronounced for given types of ACS, such as unstable angina, and in the first half of the lockdown, as the rates then started to rise again within its second half. As the incidences of weekly hospitalisations registered in the latter part of 2020 did not exceed those of 2019, the curves do not highlight the presence of a compensatory rebound in hospitalisations.

Worldwide, several studies have explored the decrease in ACS admissions during the lockdown and have suggested possible hypothesis and explanations. The first hypothesis is that many ACS did not reach the hospital, a first explanation being that many patients may have forgone hospital care for various reasons, including the fear of being infected by COVID-19 at the hospital, the concern about overloading the health services, or the willingness to avoid long waiting times. In a crisis context where the messages spread by the authorities urged people to stay home [2]. In France, during the first month of lockdown, this hypothesis can be supported by a larger reduction in hospital admissions in elderly patients or patients with STEMI; the first being fragile and therefore cautious, the second experiencing less severe symptoms and therefore being more likely to minimise them [3]. Our data do not highlight differences in this reduction according to the type of myocardial infarction, this difference possibly having been rebalanced during the catch-up phenomenon observed in the second half of the lockdown. However our study reveals a larger reduction in hospitalisations for unstable angina, which is the least severe and least symptomatic ACS. This could be related to a phenomenon of avoidance of care, which is easier in the case of a minor ACS, than in the case of a severe presentation. A second explanation for this decrease in admissions could be linked to the overload and disorganisation of the health care system, leading to dysfunctions, particularly in the prehospital stage, and missed opportunities to provide care. For example, in Gironde county, emergency calls for chest pain increased fivefold during the lockdown, while our data revealed a 26% drop in admissions for ACS in this county [16]. This is echoed in one region of England where, although admissions for ACS were also significantly impacted, emergency calls for MI remained unchanged [17]. A French study also reported a doubling of the average time between the onset of ACS symptoms and the first medical contact, with similar findings in other countries [2,18]. Overall, whether related to care avoidance events or pre-hospital care overload issues, some circumstances may have led to a substantial decrease in ACS admissions during the lockdown period. Thus, a decline in admission does not automatically imply a decrease in ACS occurrence, and it can be hypothesised that a share of the missing patients in hospitals experienced an ACS, but were not willing or able to reach the hospital and receive the needed care for their condition.

The second hypothesis to explain the decrease in ACS admissions is that there was a real decrease in the incidence of ACS during lockdown [19]. The absence of a rebound in hospitalisations following the lockdown seems to support this hypothesis. This could be the consequence of a significant change in living conditions and behaviours influencing cardiovascular risk (e.g., professional and physical activity, diet and consumption, sleep, anxiety); or of a change in environmental conditions with, notably, a reduction in air pollution connected with the reduction in activity during confinement. Indeed, air pollution is a recognized risk factor and a known trigger for ACS, and numerous studies highlights the relationship between transient elevations in ambient air pollutants and an increased risk of ischemic myocardial infarction [20]. In addition, the concentration of most air pollutants decreased transiently during the lockdown in France, to a greater or lesser extent depending on the geographical area and its baseline pollution level [21]. But interestingly, a study specifically investigating the relation between the hospital management of ACS during the lockdown and air pollutants found no association between an short term air cleaning effect and a concomitant reduction in invasive procedures for ACS [22]. These facts raise questions about the impact of a transient and geographically heterogeneous decrease in air pollutants on the occurrence of ACS, and how it may have interacted with the other factors considered. Finally, a last assumption to explain a potential decrease in the incidence of ACS would be the existence of a competitive risk on mortality between COVID-19 and MI [23].

Regarding the impact of the crisis on ACS management once patients were admitted to an hospital, our study found no significant variation in inpatient mortality during the lockdown.

### The role of determinants linked to social and territorial inequalities in health

Regarding the determinants of this decrease in admissions, our results suggest that, at the county level, a higher share of individuals on short-term working arrangements resulted in a larger decrease in admissions for ACS in 2020, i.e., a larger impact of the first lockdown on admissions. Some employment categories were more directly affected by part-time working than others; 24% of labourers, 18% of employees and 20% of shop keepers became part-time workers during the first lockdown, while the national mean was 15% [13]. Since we did not find other associations between the IRR variation and other determinants linked to occupational status or financial condition, we hypothesise that this result reflects a lower incidence of ACS among people placed on part-time working during the first lockdown. This could be explained first by the eviction from the workplace, as several studies documented the independent relationship between a high-demand occupation coupled with a low level of autonomy and the occurrence of MI [24,25]. In addition, intensive outdoor physical activity is associated with an increased risk of atheromatous plaque rupture leading to ACS [26]. A prolonged eviction from this environment could, thus, have reduced the excess risk connected with this exposure, as well as exposures to other occupational risks impacting health (e.g., tedious work, nightshifts, toxic substances). Once at home, partially employed individuals could have seized this opportunity to modify their lifestyles and improve their overall cardiovascular risk. However, this second interpretation must be taken with caution, as evolutions in modifiable cardiovascular risk factors during the lockdown have been observed in both favourable and unfavourable directions [27,28]. In contrast, at the county level, a high share of individuals with a high school degree increased ACS admissions in 2020, thus reducing the impact of confinement. This outcome can also be linked to occupation-related exposure at the time of the lockdown. In effect, 62.8% of individuals with this degree are working as employees or in intermediate occupations [29]. During the first lockdown, a majority of individuals in intermediate occupations continued to work for the most part at the worksite, as teleworking was uncommon. This was also the case for 73% of the employees who kept working [30]. These individuals thus continued to be exposed to occupational risks on cardiovascular health; in working conditions that likely worsened, increasing the burden of these exposures. In addition, a French study found an association between a job involving contact with the public, which is fairly common in these professions, and a deterioration of cardiovascular risk during the lockdown [31].

The last association in our study indicates that, at the county level, a higher density of acute care hospital equipment increased ACS admissions in 2020, thus reducing the impact of the first lockdown. Since our model is adjusted for the cumulative incidence of hospitalisations for COVID-19, this finding may reflect the effect of the resulting hospital overload. Indeed, counties with a lower level of acute care hospital resources were more likely to be overloaded with an equivalent number of COVID-19 patients, increasing dysfunctions in the prehospital care phase and complicating the admission of patients with ACS to the hospital. In a given county, greater hospital pressure may have also discouraged some patients from seeking care if they experienced ACS symptoms. This could have had the effect of exacerbating the foregoing of care, which was preexisting and probably more common in these territories due to the poorer availability of care.

### Strengths and limits

This study has various strengths. First, it includes data from all public and private hospitals in metropolitan France before, during and after the lockdown. To our knowledge, this is the first study to examine the socioeconomic and health care supply-related determinants of the impact of lockdown on ACS admissions in France. The seasonality of ACS was considered, and spatial approaches allowed us to capture the processes involved more accurately. Our study also presents limitations. First, the study is a retrospective, descriptive, observational study of hospitalised ACS only, with a comparison to the year 2019 only. Prehospital care pathways and individual patient characteristics were not investigated, and the multivariate analysis of the impact of the health crisis focused only on the first lockdown. Another limitation is that the inpatient admission data may present coding heterogeneities according to the practices of the hospitals, inducing potential biases [11]. Finally, as the data are aggregated at the county level, the results are subject to the modifiable area unit problem of scale and their interpretation to an ecological bias [14,30].

## Conclusions

This study highlights the significant impact of lockdown on ACS admissions in France, as well as the spatial variation of this impact. This variation was explained by socioeconomic and health care determinants, highlighting the impact of occupational status and hospital care supply on the incidence of ACS admissions during the lockdown. This is likely to be the result of a real change in the incidence of ACS, as well as a decrease in the number of patients reaching the hospital, due to dysfunctions in prehospital care and the fact that some patients did not seek care. Consequently, we believe that clear and targeted messages from health authorities to high-risk communities, urging them to seek care when necessary, and increased attention to the integrity of all stages of the management of emergency care, will be essential to ensure that the health of individuals is better advised and protected and to limit the collateral damages of potential future public health crises.

## Supporting information

S1 Dataset Minimal dataset. Aggregate data used to perform figures and statistical analysis.
(XLSX)
